# Post-exercise Hot Water Immersion Elicits Heat Acclimation Adaptations That Are Retained for at Least Two Weeks

**DOI:** 10.3389/fphys.2019.01080

**Published:** 2019-08-28

**Authors:** Michael J. Zurawlew, Jessica A. Mee, Neil P. Walsh

**Affiliations:** ^1^College of Human Sciences, Bangor University, Bangor, United Kingdom; ^2^School of Sport and Exercise Science, University of Worcester, Worcester, United Kingdom

**Keywords:** heat, acclimation, decay, hot water, thermal strain, thermoregulation, running

## Abstract

Heat acclimation by post-exercise hot water immersion (HWI) on six consecutive days reduces thermal strain and improves exercise performance during heat stress. However, the retention of adaptations by this method remains unknown. Typically, adaptations to short-term, exercise-heat-acclimation (<7 heat exposures) decay rapidly and are lost within 2 weeks. Short-term protocols should therefore be completed within 2 weeks of relocating to the heat; potentially compromising pre-competition/deployment training. To establish whether adaptations from post-exercise HWI are retained for up to 2 weeks, participants completed a 40-min treadmill run at 65% 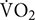
_max_ in the heat (33°C, 40% RH) before (PRE) and 24 h after (POST) the HWI intervention (*n* = 13) and then at 1 week (WK 1) and 2 weeks (WK 2) after the HWI intervention (*n* = 9). Heat acclimation involved a 40-min treadmill run (65% 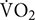
_max_) on six consecutive days in temperate conditions (20°C), followed by ≤40 min HWI (40°C). Post-exercise HWI induced heat acclimation adaptations that were retained for at least 2 weeks, evidenced by reductions from PRE to WK 2 in: resting rectal core temperature (*T*_re_, −0.36 ± 0.25°C), *T*_re_ at sweating onset (−0.26 ± 0.24°C), and end-exercise *T*_re_ (−0.36 ± 0.37°C). Furthermore, mean skin temperature (*T*_sk_) (−0.77 ± 0.70°C), heart rate (−14 ± 10 beats⋅min^–1^), rating of perceived exertion (−1 ± 2), and thermal sensation (−1 ± 1) were reduced from PRE to WK 2 (*P* < 0.05). However, PRE to POST changes in total hemoglobin mass, blood volume, plasma volume, the drive for sweating onset, sweating sensitivity and whole body sweating rate did not reach significance (*P* > 0.05). As such, the reduction in thermal strain during exercise-heat stress appears likely due to the reduction in resting *T*_re_ evident at POST, WK 1, and WK 2. In summary, 6 days of post-exercise HWI is an effective, practical and accessible heat acclimation strategy that induces adaptations, which are retained for at least 2 weeks. Therefore, post-exercise HWI can be completed during an athlete’s pre-taper phase and does not suffer from the same practical limitations as short-term, exercise-heat-acclimation.

## Introduction

Before relocating to a hot climate, athletes, military personnel and occupational workers are advised to complete 5–14 days of heat acclimation to reduce thermal strain during exercise-heat stress, improve performance, and reduce susceptibility to exertional heat illness (Cluver, 1932; Periard et al., 2015). Recommendations for heat acclimation suggest that daily exposures should elevate body temperatures (≥38.5°C) and initiate perfuse sweating through exercise-heat stress (Taylor, 2014; Periard et al., 2015; Casadio et al., 2017; Saunders et al., 2019). Hallmark heat acclimation adaptations include: an earlier onset and increase in sweating rate, a reduction in resting and exercising core body temperature, and reductions in cardiovascular strain and skin temperature that in turn, reduce perceptual strain and improve exercise capabilities in the heat (Gagge et al., 1967; Nielsen et al., 1997; Lorenzo et al., 2010; Taylor, 2014). Despite the known benefits of heat acclimation, only 15% of athlete’s heat acclimatized as part of their preparation for the hot and humid conditions in Beijing, at the 2015 IAAF World Championships (Periard et al., 2017). One explanation for poor athlete engagement with heat acclimatization is that protocols rely on sufficiently stressful natural environmental conditions in the days preceding competition to induce adaptation, which cannot be controlled or guaranteed (Gerrett et al., 2019). In addition, conventional exercise-heat-acclimation protocols are costly and impractical for non-acclimatized individuals who reside in cooler climates. Furthermore, hallmark adaptations are transient and decay quickly without regular exposure to the heat (Williams et al., 1967; Daanen et al., 2018; Pryor et al., 2019). As such, recommendations suggest that heat acclimation protocols should be completed in the days preceding relocation to a hot climate; likely compromising competition taper or pre-deployment training (Garrett et al., 2009; Saunders et al., 2019). However, completing an accessible and time efficient alternative method of heat acclimation prior to or during pre-competition taper, such as post-exercise hot water immersion (HWI) (Zurawlew et al., 2016) or sauna bathing (Scoon et al., 2007), may minimize disturbances to training and taper (Saunders et al., 2019).

Information detailing heat acclimation methods and the induced adaptations is extensive; whereas, research concerning the decay or retention of adaptation is limited (Daanen et al., 2018). Traditionally, heat acclimation decay is thought to occur rapidly (Wyndham and Jacobs, 1957; Adam et al., 1960), with 1 day of adaptation lost every 2 days spent without heat exposure (Givoni and Goldman, 1973). In addition, those adaptations that occur first, such as an expansion in plasma volume and a reduction in exercising heart rate, are thought to exhibit the most rapid decay (Williams et al., 1967; Pandolf et al., 1977; Armstrong and Maresh, 1991; Flouris et al., 2014). The number of heat exposures appears to influence the rate of decay (Daanen et al., 2018). For example, adaptations from short-term heat acclimation (<7 exposures) are typically retained for 7 days but lost after 14 days (Williams et al., 1967; Garrett et al., 2009; Neal et al., 2016). Conversely, adaptations following medium-term protocols (7–14 exposures) are in part, retained for up to 26 days (Weller et al., 2007; Daanen et al., 2011; Poirier et al., 2015). Despite heat acclimation adaptations being retained for longer following medium-term heat acclimation, short-term exercise based protocols remain the preferred preparatory method. Nevertheless, the rapid decay of adaptation from short-term heat acclimation dictates that these protocols should be completed in the 7 days preceding relocation to a hot climate. These recommendations contradict those associated with pre-competition taper, which state that accumulated fatigue should be minimized 14 days prior to competition (Mujika and Padilla, 2003; Bosquet et al., 2007). Recently, six daily exposures to post-exercise HWI was demonstrated to improve exercise performance in the heat through the initiation of heat acclimation adaptations; including, a reduction in rectal core temperature (*T*_re_) at rest and during exercise-heat stress, despite no significant changes in plasma volume or whole body sweat rate (WBSR) (Zurawlew et al., 2016). The initiation of heat acclimation was attributed to immersion in hot water, as no effect on thermoregulatory measures at rest and during exercise-heat stress was observed in a control group, who performed 6 days of submaximal exercise followed by a thermoneutral (34°C) water immersion (Zurawlew et al., 2016). Furthermore, the magnitude of adaptations from post-exercise HWI were similar in endurance trained and recreationally active individuals and compared favorably to exercise-heat-acclimation (Tyler et al., 2016; Zurawlew et al., 2018b). However, it remains unknown whether the length of adaptation retention following post-exercise HWI necessitates the completion of the protocol during a 2-week pre-competition taper and thus, suffers the same practical restraints associated with short-term exercise-heat-acclimation.

The primary aim of the current study was to assess whether adaptations are retained, 1 and 2 weeks following 6-days post-exercise HWI heat acclimation. We hypothesized that similarly to short-term exercise-heat-acclimation, adaptations would demonstrate decay at 2 weeks (Garrett et al., 2009). In addition, our previous work has suggested that post-exercise HWI does not provide a large expansion in plasma volume or increase whole body sweating rate (Zurawlew et al., 2016); adaptations commonly considered as hallmarks of heat acclimation. As such, the secondary aim of the current study was to provide a more comprehensive appraisal of blood compartment and sweating adaptations, using the optimized carbon monoxide (CO) rebreathing technique and esophageal core body temperature (*T*_es_) to measure the sensitivity and drive for sweating.

## Materials and Methods

### Participants

Thirteen recreationally active males (mean ± SD, age: 23 ± 3 years; body mass: 74 ± 7 kg; 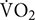
_max_: 58 ± 9 mL⋅kg^–1^⋅min^–1^) provided written informed consent to participate in the current study. All participants were healthy, non-smokers, free from any known immune, cardiovascular or metabolic diseases, and were not taking any medication. Participants had also not been regularly exposed to hot environmental conditions (including saunas and hot baths) in the 3-months prior to commencing testing. This study was received approval from the Bangor University Ethics Committee and was conducted in accordance with the Declaration of Helsinki (2013).

### Study Design

To confirm the induction of heat acclimation, participants completed an experimental trial before (PRE) and after (POST) the daily intervention. Four participants were removed from the study after the POST experimental trial as they did not comply with the study protocol. To assess the retention of adaptations, the remaining nine participants completed an experimental trial 1 week (WK 1) and 2 weeks (WK 2) after the intervention. The heat acclimation intervention involved a 40-min submaximal treadmill run in temperate conditions followed by ≤40-min HWI (40°C) completed on six consecutive days. As we have previously demonstrated no thermoregulatory benefit of a 6-day post-exercise thermoneutral water immersion (34°C: to control for training and/or hydrostatic effects), we deemed it unnecessary to include this additional control group (Zurawlew et al., 2016).

### Preliminary Measurements


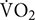
_max_ was assessed in temperate conditions (20°C) using a continuous incremental exercise test on a motorized treadmill (HP Cosmos Mercury 4.0, Nussdorf-Traunstein, Germany), as described previously (Fortes et al., 2013). The running speed that elicited 65% 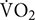
_max_ was then determined and verified using the interpolation of the running speed – 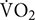 relationship. The individualized running speed was used for the submaximal running performed during each experimental trial and during the daily exercise prior to HWI. As endurance training can induce partial heat acclimation, participants were instructed to reduce their regular training by the duration completed in the laboratory. Physical activity time (>3 METS) was assessed for the duration of the study using an activity tracker (Fitbit Flex, San Francisco, CA, United States).

### Experimental Trials

Twenty-four hours prior to, and on the day of the experimental trials participants were instructed to not consume any alcohol, diuretics or caffeine, and to refrain from any additional exercise. Participants completed a diet diary in the 24 h prior to the first experimental trial (PRE) and were asked to replicate this dietary intake prior to all experimental trials (POST, WK 1, and WK 2). As sleeping patterns can influence thermoregulation (Sawka et al., 1984), participants were instructed to sleep between 2200 h and 0700 h to ensure a similar circadian pattern prior to each experimental trial. An Actigraph (Actigraph GT3X Version 4.4.0, Actigraph, Pensacola, FL, United States) was worn on the non-dominant arm with epoch length set to 1 min to analyze participants sleep quality and sleep duration using Actilife + Sleep Version 6 (Actigraph, Pensacola, FL, United States).

On the day of each experimental trial, participants arrived at the laboratory at 0730 h and were provided with a standardized breakfast (0.03 MJ⋅kg^–1^; 0.2 g⋅kg^–1^ protein, 1.0 g⋅kg^–1^ CHO, and 0.3 g⋅kg^–1^ fat) and a bolus of water equivalent to 7 mL⋅kg^–1^ of body mass. At 0800 h participants completed 20-min seated rest, dressed in t-shirt, running shorts, socks and trainers, in temperate conditions (20°C). A venous blood sample was taken without stasis and assessed for hemoglobin (Hb) concentration and hematocrit percentage. In addition, total Hb mass, blood volume, and plasma volume was assessed using the optimized CO rebreathing technique (in a subsample; *n* = 9). A mid-flow urine sample was analyzed for urine specific gravity using a handheld refractometer (Atago Uricon-Ne refractometer, NSG Precision cells, New York, United States) to confirm participants were hydrated (urine specific gravity < 1.03) (Armstrong, 2005). If participants did not meet the hydration criteria (*n* = 1), they were provided with a 500 mL bolus of water and urine specific gravity was reanalyzed; exercise began only when urine specific gravity < 1.03. A rectal thermistor and an esophageal thermistor (in a subsample; *n* = 8) was fitted, which measured rectal (*T*_re_) and esophageal temperature (*T*_es_) continuously throughout the experimental trial. A pre-exercise nude body mass was recorded using a digital platform scale (Model 705; Seca, Hamburg, Germany) and the participants were instrumented for the exercise protocol. To establish baseline measures, participants rested for a further 30 min in temperate conditions (20°C).

At 0945-h, dressed in running shorts, socks and trainers, the participant entered the environmental chamber (Delta Environmental Systems, Chester, United Kingdom) which was maintained at 33°C, 40% RH, and completed a 40-min treadmill run at 65% 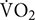
_max_ (1% gradient). During this time, no fluids were consumed; *T*_re_, *T*_es_, skin temperatures, and heart rate were monitored continuously; and rating of perceived exertion (RPE; 6–20 scale) (Borg, 1970) and thermal sensation (1–13 scale) (Hollies and Goldman, 1977) were recorded every 10 min. Local forearm sweating rate was measured every 20 s to assess the drive for sweating onset and sweating sensitivity, as previously described (Cheuvront et al., 2009). Oxygen uptake (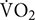), and respiratory exchange ratio (RER) were assessed from 60-s expired gas samples collected by Douglas bag method immediately prior to the 10th, 20th, 30th, and 40th min of exercise. On completion of the experimental trial participants exited the environmental chamber and rested for 15 min in temperate conditions, at which point a nude body mass was recorded to estimate WBSR. Participants remained in the laboratory until their *T*_re_ ≤ 38.5°C, during which they consumed a bolus of water equivalent to their sweat losses.

### Post-exercise HWI Heat Acclimation

Post-exercise HWI heat acclimation was completed on six consecutive days, as previously described (Zurawlew et al., 2016). During the intervention, participants were instructed to consume their normal diet and fluid intake, including caffeine and alcohol (≤3 units per day) and to report to the laboratory on six consecutive days between 0600 h and 0830 h. Prior to exercise, a nude body mass was taken and participants were fitted with a rectal thermistor and heart rate monitor. *T*_re_ and heart rate were continually monitored throughout the submaximal exercise and HWI. Dressed in running shorts, socks and trainers, participants ran for 40 min at 65% 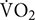
_max_ (1% gradient) on a motorized treadmill in a temperate environment (20°C). A 5 mL⋅kg^–1^ of body mass bolus of water was consumed in the first 20 min of exercise. At the cessation of exercise, dressed in shorts, participants began a semi-recumbent HWI (2–3 min transition), and submerged to the neck in a temperate room (≈ 20°C). The water was maintained at 40°C for the duration of the immersion. Immersion ended after 40 min unless the participant removed them self due to discomfort or *T*_re_ exceeded 39.9°C. Upon removal from the HWI, participants rested in temperate conditions (20°C) for 15 min, at which point a nude body mass was recorded to estimate WBSR. Participants remained in the laboratory until their *T*_re_ ≤ 38.5°C.

### Monitoring Heat Acclimation Retention

During the 2 weeks following the 6-day post-exercise HWI intervention, participants were permitted to shower but instructed to avoid significant heat exposure, including exercising in hot conditions and taking hot baths or saunas. In addition, participants were instructed to maintain their normal diet and fluid intake, including caffeine and alcohol (≤3 units per day), whilst resuming their normal exercise routine. Participants completed an experimental trial WK 1 and WK 2 after the POST experimental trial; adopting identical procedures to the PRE and POST trial.

### Measurement and Instrumentation

#### Body Temperatures

Rectal core temperature and esophageal core body temperature and were measured using a flexible, sterile, disposable thermistor (Henleys Medical Supplies Ltd., Herts, United Kingdom) and recorded using a data logger (YSI model 4000A, YSI, Dayton, United States). The rectal thermistor was inserted 10 cm beyond the rectal sphincter. The esophageal thermistor was inserted through the nasal fossae to a depth of 25% of the participant’s height (International Organization for Standardization, 2004). To assess cumulative hyperthermia, an area under the curve analysis (AUC) was performed on the daily *T*_re_ (>38.5°C) during the intervention and recovery (≈ 25 min), as previously described (Cheuvront et al., 2008). Skin thermistors (Grant EUS-U, Cambridge, United Kingdom) were attached to the right side of the body (on the chest at a midpoint between the acromion process and the nipple, the lateral mid-bicep, the anterior mid-thigh, and lateral calf) and recorded using a portable data logger (Grant SQ2020, Cambridge, United Kingdom). Mean skin temperature (*T*_sk_) was calculated using a four-site weighted equation (Ramanathan, 1964).

#### Sweating Responses

Local forearm sweating rate was measured by dew point hygrometry during all experimental trials as previously described (Fortes et al., 2013). Core temperature (*T*_re_ and *T*_es_) at sweating onset and sweating sensitivity using *T*_es_ were calculated by plotting individual relationships between local forearm sweating rate and core temperature as previously described (Cheuvront et al., 2009). Changes in dry nude body mass were used to estimate WBSR during all experimental trials and intervention days.

#### Blood Sample Collection and Analysis

Following a 20-min seated rest, venous blood samples (6-mL) were collected from an antecubital vein without stasis into an EDTA vacutainer (BD, Oxford, United Kingdom). Aliquots of whole blood were used for the immediate determination of Hb concentration (g.dL^–1^) in duplicate (Hemocue, Sheffield, United Kingdom) and hematocrit in triplicate (capillary tube method).

#### Optimized CO Rebreathing Technique

Total Hb mass (g), blood volume (mL) and plasma volume (mL) were determined in accordance with the optimized CO rebreathing technique (Schmidt and Prommer, 2005). Following a 20-min seated rest, participants were instructed to inhale 0.8–1.0 mL⋅kg^–1^ bolus of CO (99.9%), followed by rebreathing a 3 L O_2_ bolus (99.5%) using a closed glass spirometer. Prior to the CO rebreathing procedure and 4 min after, participant’s exhaled to residual volume into a CO gas meter (Drager Pac 3500, Pittsburgh, PA, United States). Prior to, 6 min and 8 min after the rebreathing procedure, earlobe capillary blood samples were assessed for carboxyhaemoglobin concentration (% COHb; ABL80 CO-OX Flex hemoximeter Radiometer; Copenhagen, Denmark). Total Hb mass was used to determine blood volume [(Hb mass/Hb concentration) × 100], red blood cell volume [mL; blood volume × (hematocrit/100)] and plasma volume (blood volume – red blood cell volume). Before commencing data collection, a preliminary reliability study (*n* = 9; triplicate measures) confirmed that the experimenter typical error of measurement was ±2.0% for total Hb mass, ±3.2% for blood volume and ±5.1% for plasma volume.

### Statistical Analysis

A sample size estimation (G^∗^Power 3.1.2) with an alpha level of 0.05 and power of 0.8, determined that nine participants were required to detect a significant reduction in end-exercise *T*_re_ (0.3°C) following short-term heat acclimation. To ensure adequate power and allowing for dropout, 13 participants were recruited. Data is presented as mean and standard deviation (SD) and statistical significance was accepted at *P* < 0.05. All data were checked for normality and sphericity. A paired sample *t-*test was used to assess for differences in thermoregulatory variables, heart rate, HWI time and AUC during the intervention (day 1 vs. day 6), and the induction of heat acclimation adaptations (PRE vs. POST; *n* = 13). A two-way repeated measures analysis of variance (ANOVA), with Greenhouse Geisser correction to the degrees of freedom (where necessary), was used to compare adaptations (e.g., end-exercise *T*_re_) to post-exercise HWI with a previous control intervention (Zurawlew et al., 2016). A one-way repeated measures ANOVA with Greenhouse Geisser correction to the degrees of freedom (where necessary) was used to assess the induction and retention of heat acclimation adaptations (PRE, POST, WK 1 and WK 2; *n* = 9). When a main effect of time was observed, results were followed up using Tukey’s *post hoc* comparison. For non-parametric data (resting *T*_re_), a Friedman test was used to assess the induction and retention of heat acclimation adaptations. When a statistical significance was found, a Wilcoxon Signed Rank tests was used to identify where the difference occurred. The magnitude of effect was reported using Cohen’s *d*, where 0.2, 0.5, and 0.8 represent small, medium and large effects, respectively (Cohen, 1988). Pearson’s correlations were used to determine the strength of the relationship between the reduction in resting and end-exercise *T*_re_ and total AUC during the heat acclimation intervention. All data was analyzed using SPSS version 24 (IBM Corporation, NY, United States), or GraphPad Prism Version 5.02 (GraphPad Software Inc., La Jolla, United States).

## Results

### Post-exercise HWI Intervention

All 13 participants completed a 40-min submaximal treadmill run followed by HWI (≤40 min) on six consecutive days. During the 6-day intervention, the endogenous stimulus for adaptation was maintained with a similar change in *T*_re_ and AUC between day 1 and day 6 of the daily intervention (*P* > 0.05; Table 1). The stimulus for adaptation was maintained due to an increase in HWI duration (*P* < 0.05; Table 1). For example, on day 1, 10 of the 13 participants removed themselves from the HWI due to discomfort; whereas, on day 6, all participants completed the 40-min HWI protocol (Table 1).

**TABLE 1:** The influence of 40-min submaximal running at 65% 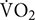
_max_ in temperate conditions followed by post-exercise hot water immersion in 40°C water to the neck (*n* = 13) on thermoregulatory variables, heart rate, and immersion time.

	**Day 1**	**Day 6**
**Submaximal exercise**		
Change in *T*_re_ (°C)	1.17 ± 0.25	1.08 ± 0.29
End-exercise *heart* rate (beats⋅min^–1^)	153 ± 11	143 ± 10^∗∗^
**Hot water immersion**		
End-immersion *T*_re_ (°C)	39.34 ± 0.30	39.24 ± 0.30
Change in *T*_re_ (°C)	0.87 ± 0.28	0.98 ± 0.15
Immersion time (min)	33 ± 7	40 ± 0^∗∗^
*n* completing 40 min immersion	3 of 13	13 of 13
**Submaximal exercise and hot water immersion**		
WBSR (L⋅h^–1^)	0.97 ± 0.29	1.09 ± 0.27^∗∗^
AUC (°C⋅min^–1^)	27 ± 16	25 ± 13

**T*_*re*_, rectal core temperature; WBSR, whole body sweat rate; AUC, area under the curve. ^∗∗^*P* < 0.01 day 6 different to day 1. Data displayed as mean ± SD.*

### Heat Acclimation Adaptations

Prior to the PRE and POST experimental trials, sleep duration (7 ± 1 h), sleep efficiency (87 ± 9%), and USG (1.018 ± 0.009) were similar (*P* > 0.05). Heat acclimation adaptations were achieved following post-exercise HWI (PRE vs. POST; *n* = 13), including a reduction in end-exercise *T*_re_ (*P* < 0.01, *d* = 1.0). For comparison, a 6-day control intervention, described previously (Zurawlew et al., 2016), did not reduce end-exercise *T*_re_ at POST (0.00 ± 0.21°C; *P* > 0.05; *n* = 7). Six days of post-exercise HWI also initiated reductions in: resting *T*_re_ (*P* < 0.01, *d* = 1.3); *T*_re_ at sweating onset (*P* < 0.01, *d* = 1.1); end-exercise heart rate (*P* < 0.01, *d* = 1.0); end-exercise PhSI (*P* < 0.01, *d* = 1.0); end-exercise *T*_sk_ (*P* < 0.01, *d* = 1.0); end-exercise RPE (*P* < 0.01, *d* = 0.9); end-exercise thermal sensation (*P* < 0.05, *d* = 0.9); mean 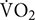 (*P* < 0.01, *d* = 0.3); and mean energy expenditure (*P* < 0.01, *d* = 0.4). No PRE to POST differences were observed for *T*_re_ – *T*_sk_ gradient, WBSR or mean RER (*P* > 0.05).

**FIGURE 1:**
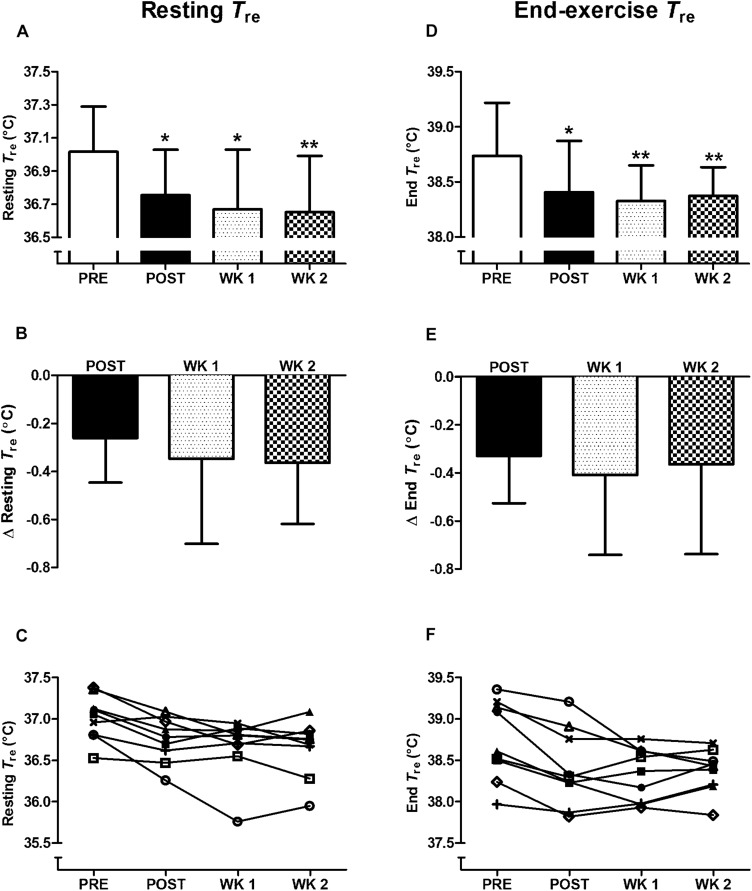
Influence of heat acclimation by post-exercise hot water immersion on resting rectal core temperature (*T*_re_) **(A–C)** and end-exercise *T*_re_ **(D–F)** following 40-min treadmill running at 65% 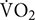
_max_ in the heat (33°C, 40% RH). Bars show mean ± SD **(A,D)** or mean ± SD of the change from PRE, at POST, WK 1, and WK 2 **(B,E)**. Lines represent individual responses **(C,F)**. ^∗^*P* < 0.05, ^∗∗^*P* < 0.01 less than PRE.

**FIGURE 2:**
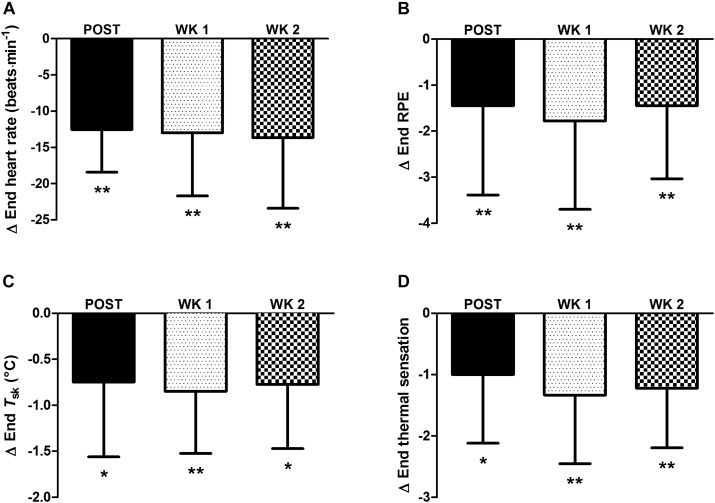
Influence of heat acclimation by post-exercise hot water immersion on end-exercise heart rate **(A)**, end-exercise rating of perceived exertion (RPE) **(B)**, end-exercise mean skin temperature (*T*_sk_) **(C)** and end-exercise thermal sensation **(D)** following 40-min treadmill running at 65% 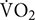
_max_ in the heat (33°C, 40% RH). Bars show mean ± SD of the change from PRE, at POST, WK 1, and WK 2. ^∗^*P* < 0.05, ^∗∗^*P* < 0.01 less than PRE (*post hoc* effect).

For completeness, in the nine participants who completed the 2-week retention protocol a main effect of time (*P* < 0.05) was observed in heat acclimation adaptations. Whereby, similar to the thirteen participants above, reductions from PRE to POST (*P* < 0.05) were noted in: resting *T*_re_ (Figure 1); *T*_re_ at sweating onset; end-exercise *T*_re_ (Figure 1); end-exercise heart rate (Figure 2A); end-exercise PhSI; end-exercise *T*_sk_ (Figure 2C); end exercise RPE (Figure 2B); end-exercise thermal sensation (Figure 2D); mean 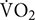; and mean energy expenditure. Of the nine participants who completed the retention protocol, seven experienced a reduction in resting *T*_re_ at POST (Figure 1C). End-exercise *T*_re_ was reduced in all nine participants at POST (Figure 1F); and end-exercise heart rate and end-exercise *T*_sk_ were lower in eight of the nine participants (Supplementary Material A,B). No main effect of time was observed for end-exercise *T*_re_– *T*_sk_ gradient, WBSR or mean RER (*P* > 0.05).

### Heat Acclimation Retention

Heat acclimation adaptations following post-exercise HWI were retained for 2 weeks; contrary to our expectation. The retention of heat acclimation was evidenced by the reduction in resting *T*_re_ from PRE to POST (*P* < 0.05, *d* = 1.0), PRE to WK 1 (*P* < 0.05, *d* = 1.1), and PRE to WK 2 (*P* < 0.01, *d* = 1.2; Figure 1). Furthermore, the reduction in thermal strain following exercise-heat stress was also retained; with a reduction in end-exercise *T*_re_ observed from PRE to POST (*P* < 0.05, *d* = 0.7); PRE to WK 1 (*P* < 0.01, *d* = 1.0) and PRE to WK 2 (*P* < 0.01, *d* = 1.0; Figure 1). At WK 2 a reduction in resting *T*_re_ (vs. PRE) was observed in eight of the nine participants. The reduction in end-exercise *T*_re_ (vs. PRE) was retained in seven of the nine participants at WK 2 (−0.52 ± 0.25°C). The two participants who demonstrated decay were among the smallest responders to the intervention at POST. Of additional interest, following 7–14 days without exposure to daily heat stress, there was a trend for the magnitude of reduction in resting *T*_re_ to increase from POST to WK 1 and WK 2 (*P* > 0.05, Figure 1). As such, to observe the full extent of the adaptive benefits, an extended period of recovery may be necessary after the post-exercise HWI intervention. Furthermore, the reduction in resting *T*_re_ at WK 2 appears to drive the reduction in end-exercise *T*_re_ (Pearson’s correlation*, r* = 0.69, *P* < 0.05). The thermal stimulus during the post-exercise HWI intervention also appeared to influence the magnitude of reduction in resting and end-exercise *T*_re_ at WK 2. Correlation analyses suggest that a greater benefit in these adaptations was observed in those who experienced a greater thermal stimulus (total AUC) during the post-exercise HWI intervention (resting *T*_re_; *r* = −0.53, *P* = 0.15, end-exercise *T*_re_; *r* = −0.48, *P* = 0.19); albeit, these correlations were non-significant.

Other heat acclimation adaptations were retained 2 weeks after the cessation of post-exercise HWI. Thus, reductions from PRE to WK 2 were observed for measures of: *T*_re_ at sweating onset (*P* < 0.01, *d* = 0.8); and end exercise heart rate (in eight of nine participants; *P* < 0.01, *d* = 0.9; Figure 2A and Supplementary Material A); PhSI (*P* < 0.01, *d* = 1.0); *T*_sk_ (in eight of nine participants; *P* < 0.01, *d* = 1.3; Figure 2C and Supplementary  Material B); RPE (*P* < 0.05, *d* = 0.9; Figure 2B); and thermal sensation (*P* < 0.01, *d* = 1.4; Figure 2D). However, the observed reductions from PRE to POST in mean 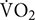 and mean energy expenditure were not observed at WK 1 or WK 2 (*P* > 0.05). The retention of heat acclimation was demonstrated despite a reduction in weekly activity time during the 2-week retention protocol (7 ± 3 h per week) compared to that during 6 days of post-exercise HWI (10 ± 4 h per week; *P* < 0.05, *d* = 0.9).

### Influence of Post-exercise HWI on Blood Compartment and Sweating Responses

**FIGURE 3:**
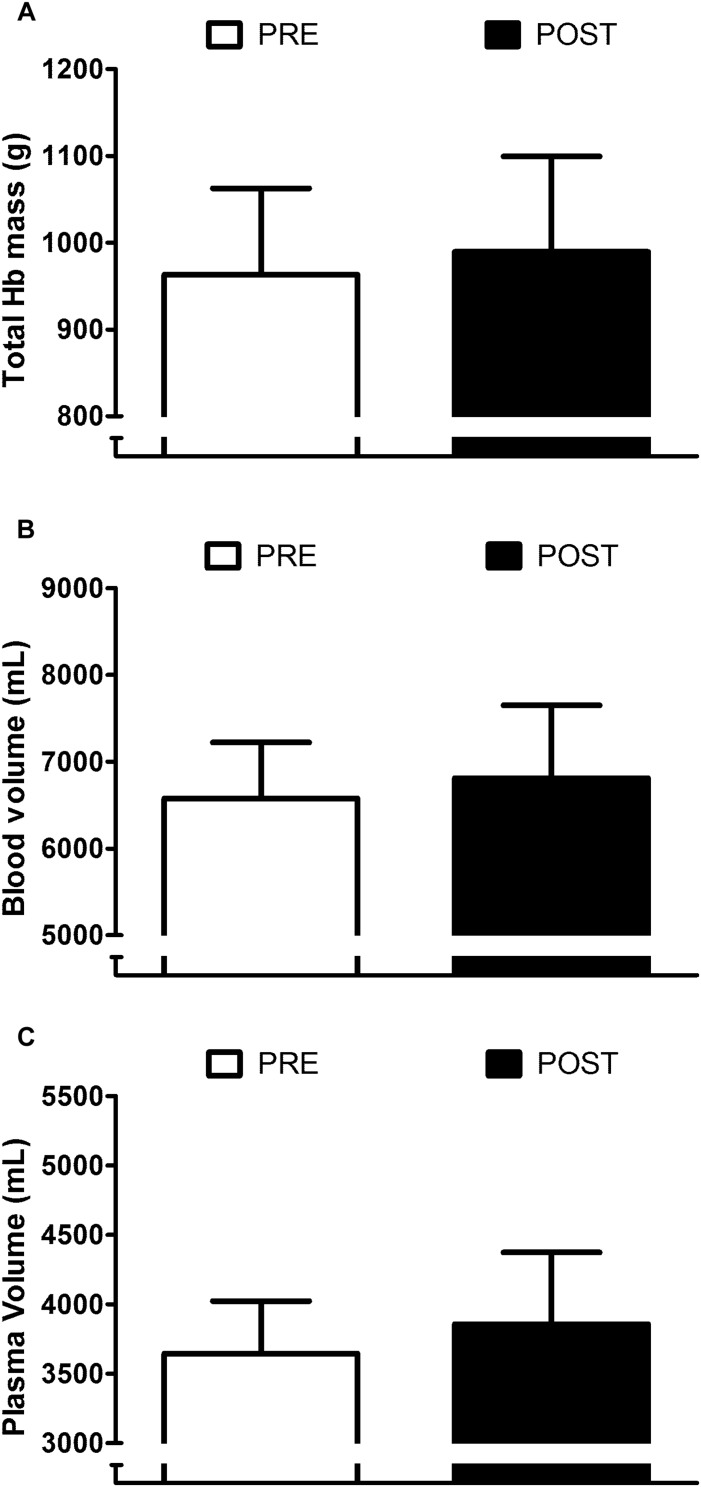
Influence of heat acclimation by post-exercise hot water immersion on total hemoglobin (Hb) mass **(A)**, blood volume **(B)** and plasma volume **(C)** at rest. Bars show mean ± SD at PRE and POST.

**FIGURE 4:**
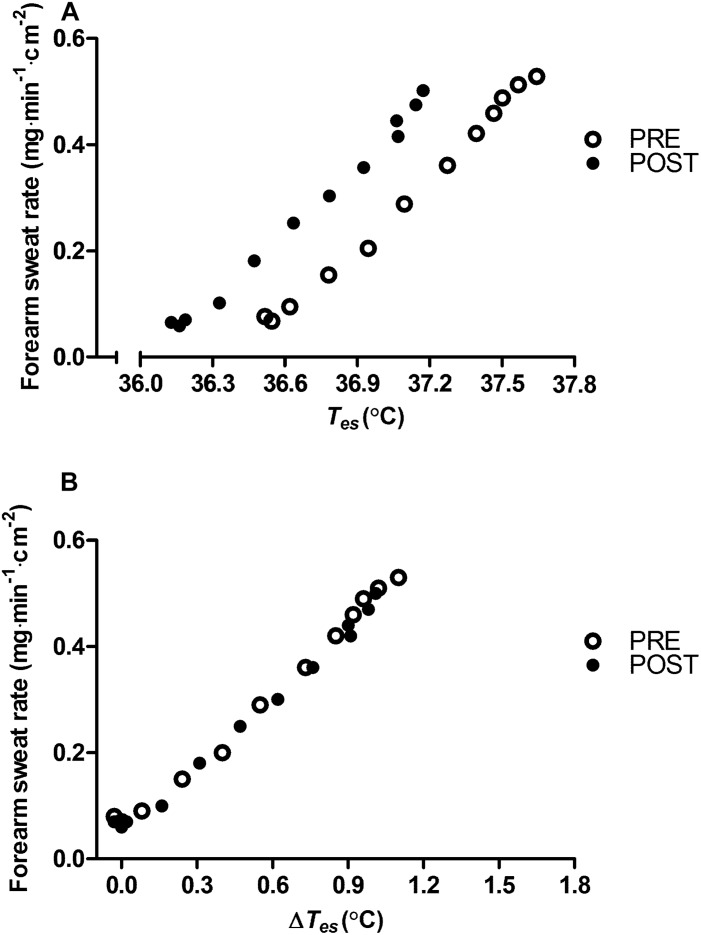
Influence of heat acclimation by post-exercise hot water immersion on mean local forearm sweating rate reported as a function of esophageal core temperature (*T*_es_) **(A)** and as a function of the change in esophageal core temperature (Δ *T*_es_) **(B)** during 12 min (1 min average) of submaximal treadmill running (65% 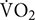
_max_) in the heat (33°C, 40% RH) before (PRE) and after (POST) heat acclimation. Error bars removed for clarity.

Appraisal of blood compartment changes demonstrate that six daily exposures to post-exercise HWI did not provide significant PRE to POST increases in: resting total Hb mass (+3 ± 5%; *P* > 0.05, *d* = 0.25; Figure 3A); blood volume (+4 ± 6%; *P* > 0.05, *d* = 0.32; Figure 3B); and plasma volume (+ 6 ± 9%; *P* > 0.05, *d* = 0.47; Figure 3C). Indeed, the effect of the 6-day intervention on plasma volume was variable, with only five of the nine participants experiencing an expansion greater than the *a priori* determined experimenter error. Examination of the influence of post-exercise HWI on sweating responses suggest that, in line with *T*_re_ at sweating onset, *T*_es_ at sweating onset during exercise-heat stress reduced from PRE to POST (−0.34 ± 0.17°C, *P* < 0.01, *d* = 1.0). However, no other elements of the sweating response altered; with no PRE to POST change in sweating sensitivity (PRE, 0.52 ± 0.23 mg⋅min^–1^⋅cm^–2^; POST, 0.59 ± 0.31 mg⋅min^–1^⋅cm^–2^; Figure 4A) or the drive for sweating onset (delta change in *T*_es_; PRE, 0.38 ± 0.11°C; POST, 0.42 ± 0.21°C; Figure 4B).

## Discussion

The present study is the first to examine the retention of heat acclimation adaptations following 6 days of post-exercise HWI. In accordance with our previous findings, post-exercise HWI induced heat acclimation adaptations in all participants (Zurawlew et al., 2016). Contrary to our hypothesis, and the notion that adaptations following short-term exercise-based protocols decay by 2 weeks, the new and noteworthy findings provide evidence of the retention of heat acclimation for at least 2 weeks following the post-exercise HWI intervention. For example, a similar extent of adaptation at POST, WK 1, and WK 2 was observed for measures of resting *T*_re_, *T*_re_ at sweating onset, and end-exercise *T*_re_, heart rate, *T*_sk_, RPE and thermal sensation (Figures 1, 2); despite no significant changes in plasma volume or WBSR. The induction and retention of heat acclimation is likely due to the large daily elevations in both core (change in *T*_re_ ≈ 2.1°C) and skin temperatures (*T*_sk_ = 40°C) during immersion in hot water to the neck. We have confidence that immersion in hot water provides these adaptations, as we have previously demonstrated no thermoregulatory benefits from 6 days of submaximal exercise and immersion in thermoneutral water (Zurawlew et al., 2016). Exposure to the dual thermal stimulus during immersion in hot water (elevated core and skin temperature) appears to initiate the reduction in resting core temperature; attenuating thermal strain during exercise-heat stress for at least 2 weeks following the HWI intervention. Recommendations state that a 2-week pre-competition taper with a reduction in training volume (≈ 50%) optimizes athletic performance (Bosquet et al., 2007); although, smaller reductions in training volume (15–20%) 7 days from competition have yielded successful performances in elite athletes (Tonnessen et al., 2014; Solli et al., 2017). The retention of adaptation from post-exercise HWI for at least 2 weeks indicates that taking a hot bath after training in temperate conditions can be completed during the pre-taper phase, without compromising supercompensatory adaptations (Tonnessen et al., 2014).

The retention of heat acclimation for at least 2 weeks following post-exercise HWI appears favorable compared to short-term exercise-heat-acclimation and aligns more closely with the retention timeframe following medium-term protocols (Weller et al., 2007; Garrett et al., 2009; Daanen et al., 2011). However, despite evidence of heat acclimation retention 12–26 days following medium-term protocols, a portion of the induced adaptation is lost (20–35%) (Pandolf et al., 1977; Weller et al., 2007). A recent meta-analysis suggested that adaptation decay is dependent upon extent of induced adaptation and estimated a rate of decay of ≈ 2.5% every day without exposure to the heat (Daanen et al., 2018). Accordingly, the two participants who gained the smallest benefit from post-exercise HWI at POST demonstrated decay at WK 2. Surprisingly, there was no evidence of adaptation decay after 2 weeks in seven of the nine participants (end-exercise *T*_re_; −0.52°C). On the contrary, the mean data suggests a further gain in the extent of adaptation for resting (+40%) and end-exercise *T*_re_ (+11%) was observed despite no exposure to any significant heat stress. It is possible that the experimental trial at WK 1 provided a thermal stimulus to maintain adaptation (Pryor et al., 2019); however, the endogenous thermal stimulus during this trial was relatively low (end-exercise *T*_re_, 38.3°C). Therefore, the favorable retention of adaptations following post-exercise HWI is more likely due to the large daily elevations in core and skin temperatures during immersion to the neck in hot water. Exposure to a large dual thermal stimulus augments the magnitude of adaptation and provides benefits including: a reduction in resting and exercising core temperature, that decay at a slower rate (Pandolf et al., 1977; Weller et al., 2007). Whereas, widely regarded hallmark heat acclimation adaptations, such as an expansion in plasma volume (Armstrong and Maresh, 1991), occur following exposure to a smaller accumulative thermal stimulus and decay rapidly (Williams et al., 1967; Pandolf et al., 1977). The current data confirms the reduction in thermal strain from post-exercise HWI is initiated by the reduction in resting *T*_re_ (≈−0.3°C), not through significant changes in blood compartments or sweating responses within the conditions of our experimental trial. As previously suggested (Zurawlew et al., 2018b), the semi-recumbent body position and/or hydrostatic squeeze during HWI may limit plasma volume expansion; and a greater number of exposures may be required to initiate an increase in WBSR. Therefore, as sweating occurs following a similar rise in *T*_re_ after post-exercise HWI (Figure 4), the reduction in resting *T*_re_ likely initiates the attenuation in *T*_re_ at sweating onset and during exercise-heat stress (Zurawlew et al., 2016, 2018a, b). Furthermore, the reduction in resting *T*_re_ at POST was maintained at WK 2 (−0.36°C), supporting the notion that adaptations requiring exposure to a larger thermal stimulus are retained for longer (Williams et al., 1967; Pandolf et al., 1977; Armstrong and Maresh, 1991; Daanen et al., 2011; Flouris et al., 2014). Interestingly, the retention of the reduction in resting *T*_re_ at WK 2 (−0.36°C) appears to be fully responsible for the attenuation in thermal strain during exercise-heat stress (end-exercise *T*_re_; −0.36°C). As such, the induction of adaptations provided through exposure to large daily elevations in core and skin temperatures, such as a reduction in resting core temperature, may initiate the retention of heat acclimation for at least 2 weeks following post-exercise HWI. In addition, due to the ease of assessing resting core temperature in temperate conditions, this measurement could be utilized as a metric to outline the extent of induced adaptation and, following definition of the slope of decay, to forecast the timeframe of adaptation retention.

A reduction in resting core temperature has previously been linked to alterations in basal metabolic rate following both endurance training and seasonal heat acclimatization (Baum et al., 1976; Buguet et al., 1988). In line with these observations, a large and meaningful reduction in resting core temperature (−0.32°C) occurs following long-term exercise-heat-acclimation (≥15 exposures) (Patterson et al., 2004; Tyler et al., 2016). Whereas, both short and medium-term exercise-heat-acclimation induce a smaller attenuation in resting core temperature (≈−0.2°C), according to a recent meta-analysis (Tyler et al., 2016). Surprisingly, after six daily exposures to post-exercise HWI a large reduction in resting *T*_re_ is observed (≈−0.3°C) (Zurawlew et al., 2016, 2018a, b). Comparison of the HWI protocol with the extant exercise-heat-acclimation literature may explain the difference in the magnitude of adaptation. For example, immersion to the neck in hot water exposes individuals to a high core temperature (end-immersion *T*_re_; 39.3°C) and perhaps more importantly, to a large exogenous thermal stimulus, where skin temperatures likely equilibrate with water temperature (≤40 min, ≈ 40°C) (Zurawlew et al., 2016, 2018a, b). Whereas, exercise-heat-acclimation protocols which utilize the controlled hyperthermia technique clamp *T*_re_ at 38.5°C (Fox et al., 1963; Garrett et al., 2009) and do not elevate skin temperatures to the same magnitude as HWI. Therefore, exposure to the large dual thermal stimulus during HWI, which is considered to promote a more complete state of adaptation (Fox et al., 1964; Regan et al., 1996), may provide the large magnitude of adaptation and accelerate the occurrence of a reduction in resting core temperature. Specifically, the elevation in skin temperature during HWI to the neck may activate warm-sensitive neurons and induce hypothalamic neural network changes, which reduce resting core temperature (Tan et al., 2016; Zhao et al., 2017; Siemens and Kamm, 2018). Interestingly, the reduction in resting *T*_re_ observed at WK 1 and WK 2 was greater than that observed at POST (≈−0.1°C); thus, supporting previous work that suggests a period of recovery in cool conditions may augment the reduction in resting *T*_re_ (Daanen et al., 2011). This augmentation of adaptation may represent a delay in physiological remodeling following exposure to the heat (Horowitz, 2016) and warrants further investigation to improve the integration of heat acclimation into training programs.

Heat acclimation recommendations suggest that daily exercise-heat-stress is the most effective method for acclimating to the heat (Taylor, 2014; Periard et al., 2015). In accordance with our recent work (Zurawlew et al., 2016, 2018a, b), the present findings offer an alternative approach, by demonstrating that taking a hot bath following exercise in temperate conditions is an effective, practical, and accessible heat acclimation strategy. For example, post-exercise HWI improves endurance performance in the heat (Zurawlew et al., 2016) and induces heat acclimation adaptations (Zurawlew et al., 2018a, b), which in the majority of participants are retained for at least 2 weeks. Furthermore, incorporating a hot bath into post-exercise washing routines on 6 days reduces interference with training/taper while ensuring that athletes are adapted to the heat prior to travel for competition. As such, an athlete may be less reliant on natural acclimatization upon arrival, where environmental conditions may not be sufficiently challenging to induce adaptations (Periard et al., 2017; Gerrett et al., 2019). To ensure safety, practitioners and athletes utilizing the post-exercise HWI protocol should limit immersion duration (≤40 min), be mindful to monitor physiological measures, and terminate exposures if intolerance symptoms arise (e.g., high core temperature and/or sensations of thermal strain). We recognize that the current research does not illustrate the timeframe of decay from post-exercise HWI in all participants or directly compare the magnitude and retention of adaptation with exercise-heat-acclimation. Future research should examine the influence of the large daily elevations in skin temperature during HWI on heat acclimation adaptation. In addition, investigations should confirm the underpinning mechanisms for the retention of heat acclimation and examine the effect of post-exercise HWI on training load and recovery, whilst establishing if the maintenance of adaptations translates to performance. To improve the practicality of HWI, future studies should also establish whether meaningful adaptation and retention timeframes are observed following protocols of fewer or a greater number of HWI exposures.

## Conclusion

Heat acclimation adaptations from post-exercise HWI are retained for at least 2-weeks. As such, for athletes who reside in temperate conditions, taking a hot bath following routine training on six consecutive days during the pre-taper phase represents a simple, practical and effective heat acclimation strategy.

## Data Availability

The datasets generated for this study are available on request to the corresponding author.

## Ethics Statement

This study received ethical approval from the Ethics Committee of the School of Sport, Health and Exercise Sciences, Bangor University.

## Author Contributions

NW had primary responsibility for the final content. All authors were involved in the conception of the project and development of the research plan, performed the data analysis, interpreted the data, and prepared the manuscript. MZ led the data collection.

## Conflict of Interest Statement

The authors declare that the research was conducted in the absence of any commercial or financial relationships that could be construed as a potential conflict of interest.
